# Isolation and Composition Analysis of Bioactive Glycosaminoglycans from Whelk

**DOI:** 10.3390/md16050171

**Published:** 2018-05-18

**Authors:** Chrow Khurshid, David Alexander Pye

**Affiliations:** School of Environment and Life Sciences, University of Salford, Manchester M5 4WT, UK; d.pye@salford.ac.uk

**Keywords:** glycosaminoglycans, marine mollusca, anti-cancer, anti-proliferation, heparan sulphate

## Abstract

Glycosaminoglycans (GAGs) are found covalently attached to proteins, which create conjugates known as proteoglycans. GAGs have remarkable biological activity as co-receptors for a variety of growth factors, cytokines, and chemokines. The present study identifies the key compositional differences between the GAGs isolated from whelk and mammalian GAGs. This polysaccharide represents a new, previously undescribed GAG with cytotoxic activity on cancer cells. Disaccharides were obtained by sample digestion with heparinases I, II, and III and chondroitinase ABC. The resistant oligosaccharides from whelk GAGs treated with heparinase I, II, and III and chondroitinase ABC were retained by the filter due to their larger size. Disaccharide analysis was performed using Glycan Reduction Isotope Labeling (GRIL LCQ-MS). The amounts of filter-retained fragments, as assessed by monosaccharides analysis, suggested that a proportion of the whelk GAG chains remained resistant to the enzymes used in the disaccharide analysis. Thus, the proportions of individual disaccharide produced in this analysis may not truly represent the overall proportions of disaccharide types within the intact whelk GAGs chain. However, they do serve as important descriptors for the classification and make-up of the anti-cancer GAGs chains. Furthermore, these data represent clear evidence of the compositional differences between whelk GAGs and commercial mammalian GAGs.

## 1. Introduction

Glycosaminoglycans (GAGs) are found covalently attached to proteins, creating conjugates known as proteoglycans. GAGs are linear glycan chains composed of a repetitive structure of disaccharide units of uronic acid and glucosamine. Initially, it was thought that GAGs only existed in mammals. However, they are found in a wide variety of organisms, including fishes, insects, fungi, yeast, and bacteria. It is a particularly rich component in the muscle of shellfish [1]. Glycosaminoglycans have remarkable biological activities as co-receptors for a variety of growth factors, cytokines, and chemokines [2]. In addition, sulphated polysaccharides have been recognized to possess various biological properties, including anticoagulation, antiviral effects, and immunomodulatory activity that might find relevance in nutraceutical/functional foods, cosmetic/cosmeceutical and pharmaceutical applications [3]. Furthermore, the biological function of proteoglycans includes the regulation of cell proliferation through the interaction of the GAGs chains in proteoglycans with proteins, such as growth factors and their receptors [4].

There are many types of GAG: hyaluronic acid (HA), chondroitin sulphate (CS), dermatan sulphate (DS), keratan sulphate (KS), heparin (Hep), and—the most widely studied—heparan sulphate (HS). The main differences between the family members are the position and configuration of their glycoside linkages, the amount and location of their sulphate substituents, and their monosaccharide composition. Some of these species have a very simple structure, such as hyaluronic acid, while others, such as CS, DS, Hep and HS, contain extensive blocks of modified disaccharide units with complex sulphation patterns that appear to be under tight biosynthetic control [5]. 

Heparin and HS have been intensively studied due to their ability to bind many proteins that regulate a variety of important biological processes [6].

Heparan sulphates are comprised of alternating glucosamine and uronic acid residues. The disaccharide units in the heparin chain are extensively modified, which differs from the HS chain. In HS biosynthesis and modification, the selected GlcNAc residues likely undergo the *N*-deacetylation/*N*-sulfation modification. Thus, the HS chain appears as blocks of unmodified sequences (*N*-acetyl *N*Ac-domain), which are separated by highly modified residues of *N*-sulphate (*N*S-domain) [7].

However, in heparin, the chain essentially contains *N*-sulphate (*N*S-domain) interrupted by *N-*acetyl (*N*Ac*-*domain). During the modification reactions, three types of domains are likely to be generated. First, the unmodified *N*-acetylated regions (*N*A-domain) with glucuronic acid (GlcA) units, which are present in small amounts in the predominant disaccharide units in HS. The second type are adjacent *N*-sulphated regions with 2-*O*- and 6-*O*-sulphated iduronic acid (IdoA) and glucosamine residues respectively in the *N*S-domain. Finally, alternating regions of *N*-sulphated and *N*-acetylated disaccharides domain appear in HS chains [8,9].

GAGs have been isolated from various tissues obtained from a large number of animal species, including both vertebrates and invertebrates [10]. Heparin has only been found in one invertebrate phylum, the Mollusca, and it often constitutes up to 90% of the total GAG content of these organisms. The heparin that has been isolated from various molluscs was found to be structurally different from human heparin and pharmaceutical heparin [11,12].

Nowadays, there is an interest in developing potent therapeutic agents from non-mammalian GAG sources. Hence, these novel GAGs provide possible alternative therapeutic agents for treating diseases such as cancer [13,14].

In this study, we isolated the complex polysaccharides from the soft body of whelk. The main differences in the composition of these GAGs and mammalian GAGs occur due to the abundance of disaccharides and different sulphation patterns within the glycan chain. This whelk polysaccharide has a high anti-cancer activity and is able to inhibit the growth of several cancer cell lines, which have been tested in vitro. The anti-proliferative activity of these GAGs on triple negative breast cancer (TNBC) cell lines is reported in this study. Moreover, the anti-proliferation activity of GAGs on ER+ cells, such as MCF-7 and SKBR-3, was evaluated, and significant inhibition was reported (data not shown). The biological activity of these highly sulphated GAGs was strongly connected to their unique composition and degree of sulphation, which proved to be different from mammalian glycosaminoglycans, with no anti-cancer activity.

## 2. Results

### 2.1. Glycan Reductive Isotope Labelling (GRIL) Analysis of GAGs Disaccharides

Disaccharide analysis was performed using a GRIL LCQ-MS analysis protocol for GAG disaccharides [15]. A new shorthand nomenclature for the disaccharide subunit structure of GAGs was used in the present study according to Lu et al. [16]. The results from the disaccharide analysis of the CS and HS components of the whelk GAG chain are shown in Table 1 and Table 2, which show significant differences in the disaccharide composition and overall sulphate content in comparison to commercial porcine HS and CS (Table 3 and Table 4). ∆HexA2S-GlcNS and ∆HexA2SGlcNS6S constituted 92–93% of the *N*-sulphated disaccharides found in CK-1, compared with 61–62% present in commercial HS, in terms of individual disaccharide, which suggested that ∆HexA2S-GlcNS and ∆HexA2SGlcNS6S were enriched in the whelk GAG sample.

There is a notable difference in the disaccharide ∆HexA2S-GlcNS, which is the most abundant disaccharide in the whelk GAGs (43.15%) and the second-least abundant in the porcine heparin (0.9%). Disaccharide analysis confirmed the presence of CS chains in the whelk samples CK-1, CK-2 and CK-3, and the amount of individual CS disaccharide was determined by GRIL LCQ-MS GAGs disaccharide analysis. Chondroitinase ABC degradation of the whelk GAG sample confirmed the presence of susceptible linkages as a result of the presence of CS in the anti-cancer GAG samples. The MTT assay of whelk GAGs treated with chondroitinase ABC showed no decrease in anti-cancer activity. This is in marked contrast to the whelk GAGs treated with heparinases I, II, and III, which showed a significant loss of anti-cancer activity after sample degradation and incubation with triple negative breast cancer MDA-MB-468 and MDA-MB-231 cell lines.

### 2.2. Monosaccharide Quantitative Analysis of GAGs’ Filter-Fraction Fragments

**Figure 1:**
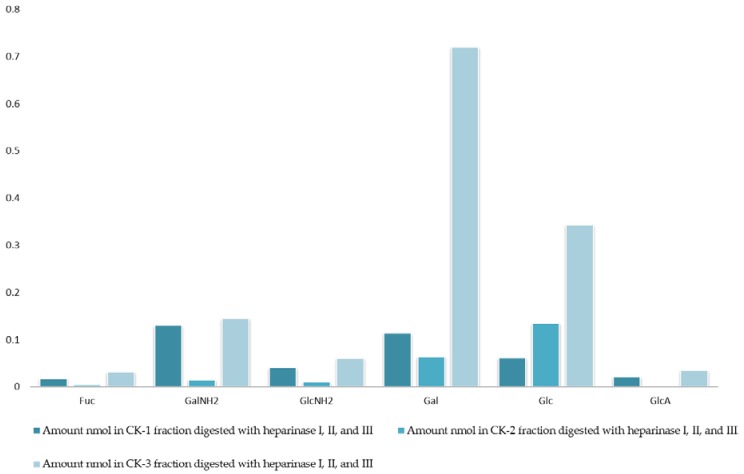
Monosaccharide composition analysis of fractions CK-1, CK-2, and CK-3. All fractions were eluted from the ion-exchange column and digested separately by heparinases I, II, and III. Fractions were further analyzed using the 10K filter (nano-centrifugal device with omega membrane).

**Figure 2:**
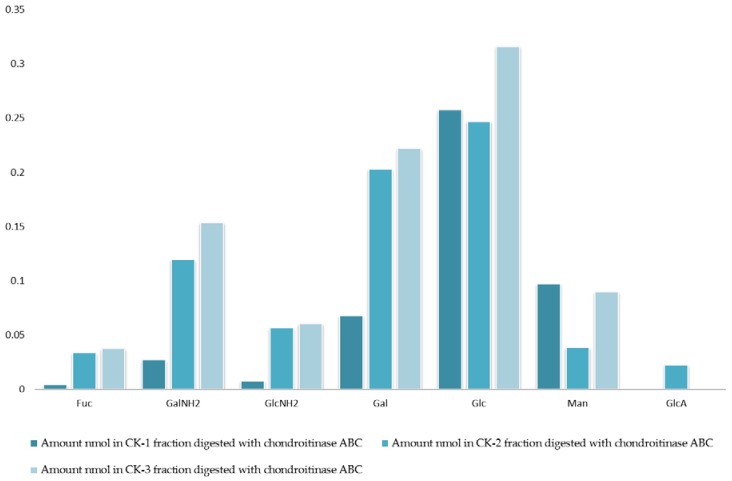
Monosaccharide composition analysis of fractions CK-1, CK-2, and CK-3. All fractions were eluted from the ion-exchange column and digested separately by chondroitinase ABC. Fractions were further analyzed using the 10K filter (nano-centrifugal device with omega membrane).

**Figure 3:**
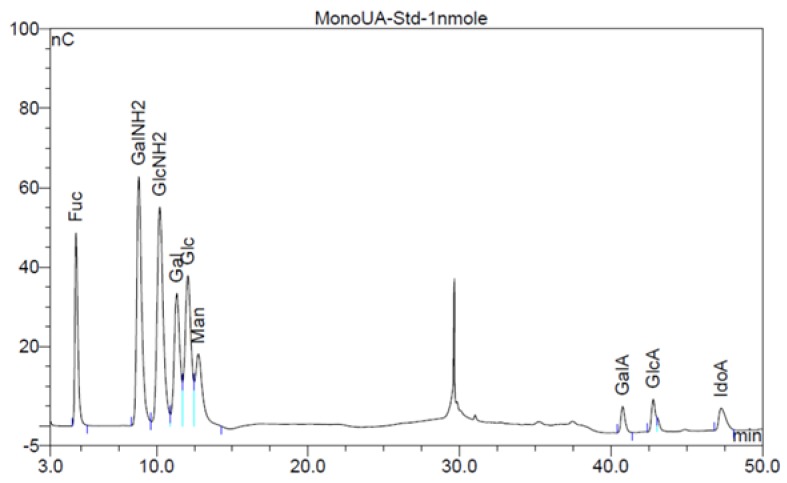
Chromatogram of reference monosaccharides.

The analysis of monosaccharides for the filter-retained fragment of CK-1, CK-2 and CK-3 was performed in order to determine the monosaccharides that remained in the mixture. The data in Figure 1 and Figure 2 show the monosaccharide compositions for CK-1, CK-2 and CK-3 filter-retained fragments from whelk GAGs in comparison to the standard monosaccharide uronic acid (MonoUA-Std) (Figure 3). The amount of filter-retained material, as assessed by monosaccharide analysis, suggested that a proportion of the whelk GAG chain remained resistant to the enzymes used in the disaccharide analysis. Furthermore, the monosaccharide analysis suggested considerably lower levels of IdoA in whelk GAGs when compared to mammalian GAGs. This suggests significant differences in the epimerization from glucuronic acid GlcA to iduronic acid IdoA in the synthesis of whelk and mammalian GAGs. The presence of considerable numbers of *O-*sulphated GlcA in whelk GAGs may be linked to the difference in anti-cancer activity.

### 2.3. Cytotoxic Activity of Intact GAGs and Their Fragments on Triple Negative Breast Cancer

**Figure 4:**
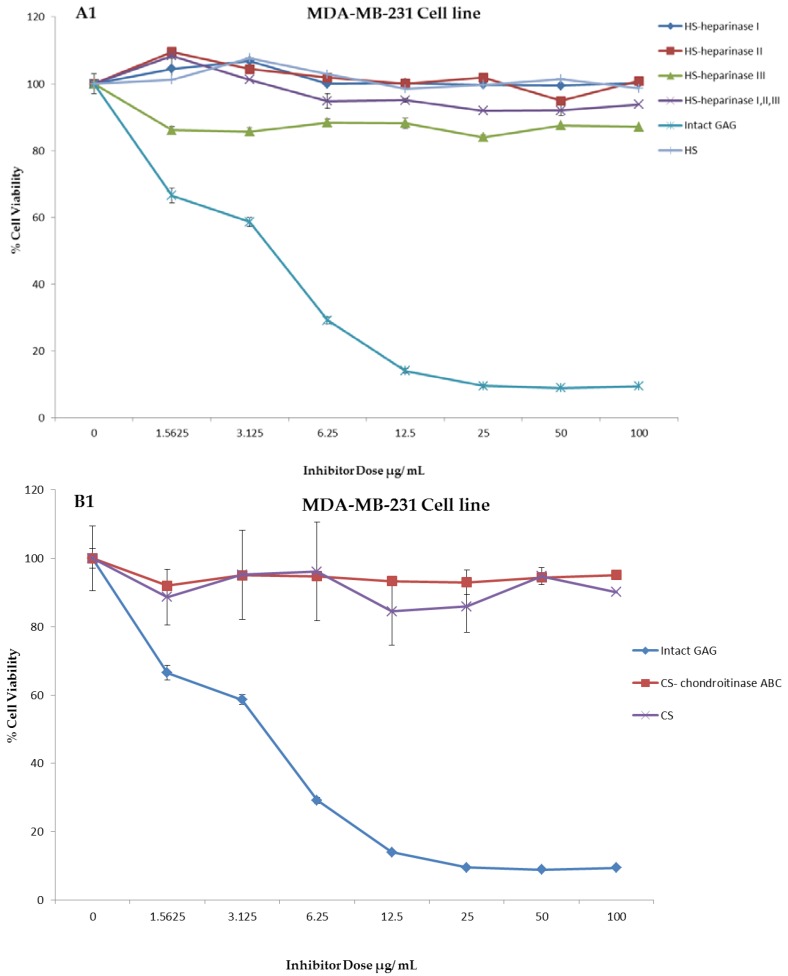

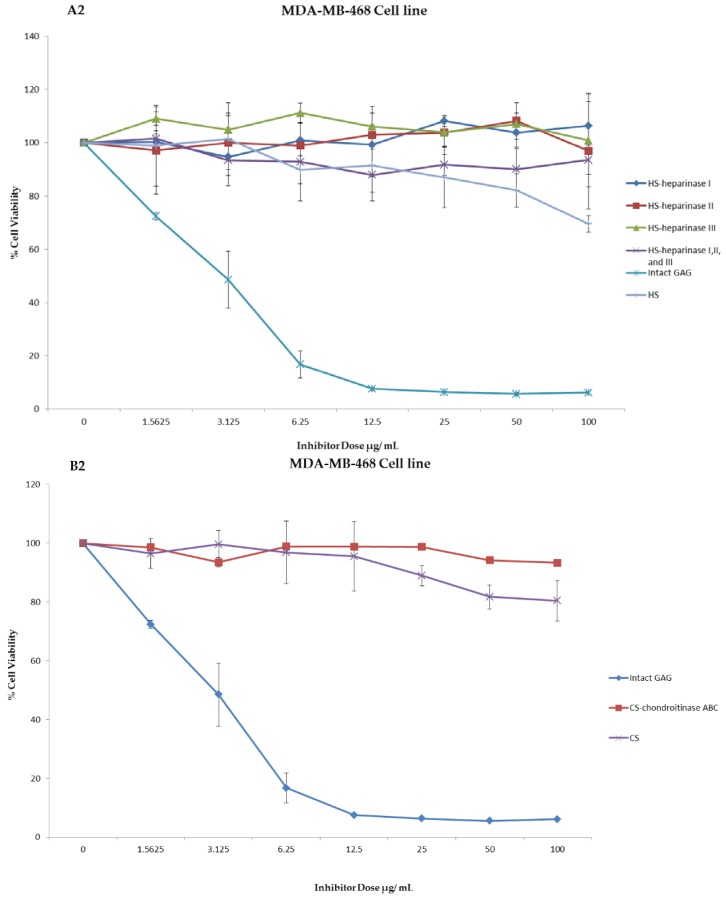
Anti-proliferation activity of crude whelk GAG extract compared to the activity of commercial mammalian HS and CS GAGs before and after enzymatic degradation. **Panels A1** and **B1**: shows the effect of whelk GAGs on the MDA-MB-231 cell line. **Panels A2** and **B2**: shows the effect of whelk GAGs on the MDA-MB-468 cell line. The data represent the percentage of viable cells for sample size (*n* = 3) as mean ± SD. Cells were cultured in monolayers and maintained at 37 °C in a humidified 5% CO_2_ atmosphere. The cytotoxicity assay was performed using the MTT colorimetric assay.

An experiment was carried out to assess the impact of the crude whelk GAG extracts and their fragments on the cell proliferation in triple negative breast cancer. Whelk GAG crude extract has significant inhibitory activity on the triple negative breast cancer MDA-MB-231 and MDA-MB-468 cell lines. Remarkable EC_50_ values were observed in both cell lines, and were approximately 3.48 ± 0.02 µg/mL and 2.76 ± 0.01 µg/mL, respectively. The bioactivity of whelk GAGs and commercial mammalian HS/CS on triple negative breast cancer cell lines were screened under two conditions: cells treated with crude whelk GAGs; and cells treated with the whelk GAGs following digestion by heparinases I, II, and III, and chondroitinase ABC. The results indicated no inhibition of growth in TNBC from commercial mammalian HS and CS, either before or after enzymatic degradation (Figure 4). In contrast, significant inhibition was observed following treatment with whelk GAG extract before and after enzymatic degradation with HS and CS lyases (Table 5). Nevertheless, the biological activity of the fragments generated from heparinases I, II, and III was slightly reduced in comparison to the fragments generated from chondroitinase ABC.

### 2.4. Identification of the GAGs’ Active Fractions for Triple Negative Breast Cancer

**Figure 5:**
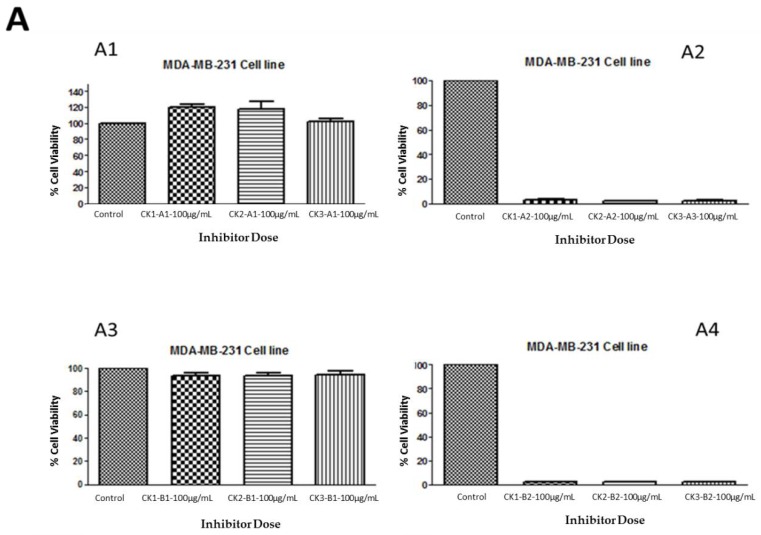

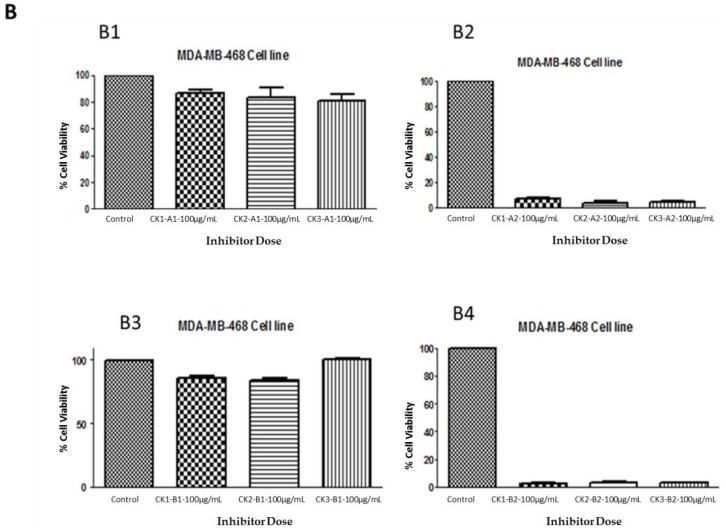
Anti-proliferation activity of whelk GAGs fractions of CK-1, CK-2 and CK-3 before and after enzymatic degradation on triple negative breast cancer cell lines. **Panel A**: Inhibition activity of FT and R fractions from heparinases I, II, and III and chondroitinase ABC fragments on the MDA-MB-231 cell line. **A1** shows the activity of FT fragments from a 10K filter of heparinases I, II, and III degradation of whelk GAGs. **A2** shows the activity of R fragments from a 10K filter of heparinases I, II, and III degradation of whelk GAGs. **A3** shows the activity of FT fragments from a 10K filter of chondroitinase ABC degradation of whelk GAGs. **A4** shows the activity of R fragments from a 10K filter of chondroitinase ABC degradation of whelk GAGs for each fraction of CK-1, CK-2 and CK-3 from the anion-exchange chromatography. **Panel B**: Inhibition activity of FT and R fragments from heparinases I, II, and III and chondroitinase ABC on the MDA-MB-468 cell line. **B1** shows the inhibitory activity of FT fragments from a 10K filter of heparinases I, II, and III degradation of whelk GAGs. **B2** shows the inhibitory activity of R fragments from a 10K filter of heparinases I, II, and III degradation of whelk GAGs. **B3** shows the inhibitory activity of FT fragment from a 10K filter of chondroitinase ABC degradation of whelk GAGs. **B4** shows the inhibitory activity of the R fragment from a 10K filter of chondroitinase ABC degradation of whelk GAGs for each fraction of CK-1, CK-2 and CK-3 from the anion-exchange chromatography. The data represent the percentage of viable cells for sample size (*n* = 3) as mean ± SD. Cells were cultured in monolayers and maintained at 37 °C in a humidified 5% CO_2_ atmosphere. Cytotoxicity assay was performed using the MTT colorimetric assay.

The crude extraction from whelk GAGs was analyzed using a variety of analytical techniques in order to identify the structure of the GAGs in relation to the active part of the polymer that is responsible for their activity on triple negative breast cancer cell lines. The inhibitory activity was retained by the filter-retained fragments (R) that did not pass through the centrifugal filtration device following the enzymatic degradation of all fractions obtained from whelk GAGs using ion-exchange chromatography. An equal amount (100 µg/mL) of flow-through (FT) and filter-retained (R) fragments from fractions (CK-1, CK-2 and CK-3) were incubated with both cell lines for five days at 37 °C and 5% CO_2_. The cells were cultured in a monolayer in 96-well plates and incubated for 24 h, at which point the medium was changed and a fresh medium was added to all wells. Cytotoxic activity was obtained using the MTT assay. The data were validated using the GraphPad Prism 5.0 software. The results shown in Figure 5 demonstrate the activity of whelk GAGs that had been degraded by heparinases I, II, and III or chondroitinase ABC and passed through the filter. These FT fragments are more likely to contain disaccharides or oligosaccharides that are smaller in size than the pore size of the filter, and are therefore capable of passing through the device. The results shown in panel A1 and panel B1 of Figure 5 indicate no effect of the FT fragments that were treated with heparinases I, II, and III on either the MDA-MB-231 or MDA-MB-468 cell lines. Moreover, no inhibitory activity on either cell line was seen for the FT fragments from chondroitinase ABC treatment, as shown in Panels A3 and B3. On the other hand, significant inhibitory activity on the cell lines was observed in the filter-retained fragments (R) of heparinases I, II, and III and chondroitinase ABC.

## 3. Discussion

Extraction and subsequent purification of GAGs from whelk showed that the soft tissue contained a large amount of GAGs. These GAGs contained highly sulphated polysaccharides compared to porcine mucosal heparin or CS. Disaccharide analysis showed the abundance of different disaccharide units present in whelk GAGs and sulphation patterns that differed from mammalian GAGs.

The partial resistance of this polysaccharide toward heparinases I, II and III suggested that this polysaccharide had a different composition to the heparin and HS from mammalian tissues [17]. In this study, small amounts of GlcA were observed, and no presence of IdoA from the filter-retained fragment was noticed, which might be associated with structural heterogeneity in these GAGs, or be due to a unique modification in the incomplete epimerization of GlcA to IdoA. This might be the main reason for their anti-cancer activity.

Ion-exchange chromatography enabled the fractionation of the GAG samples according to their net charged groups using the stepwise addition of sodium chloride with three different buffer strengths. The results show that approximately 90% of the total sample applied to the column was eluted with 1M NaCl, which was followed by smaller amounts of GAGs eluted with 2M and 3M NaCl. The results show the abundance of different disaccharide units present in whelk GAGs, along with their sulphation patterns, which differ significantly from mammalian GAGs, for the first time. Reasonable amounts of GAGs were detected from the filter-retained fragment of the Nanosep centrifugal tube containing the omega membrane (10K filter Eppendorf tube), which could also be the result of incomplete digestion by heparinases I, II, and III and chondroitinase ABC. The filter-retained fragments showed cytotoxicity toward the cancer cell lines while the flow-through was inactive. This finding was unexpected and suggested that the filter-retained fragments might contain the key heparinase-resistant sequences that might be responsible for the biological activity, and as such, elucidating their structure is important for future studies.

The presence of this polysaccharide with a simple but unique sequence raises questions about their biological activities, including their roles in cancer treatment. The administration of some glycosides leads to many cellular changes, including reduction of cancer cell adhesion, suppression of cell migration and tube formation in cancer cells, suppression of angiogenesis, inhibition of cell proliferation and tumor invasion. As a result, growth inhibition of tumors occurs in vitro and in vivo [18].

Whelk CS-GAGs may have anti-cancer activity; but the chondroitinase ABC treatment followed by the MTT assay did not support this assumption. Fucosylated CS may be present in the sample and this could conceivably be resistant to chondroitinase ABC. However, the digestion of whelk GAGs with heparinase and anti-cancer assessment of the fragments still suggests that a HS is still the primary target in the search for the active components within the whelk GAG sample.

## 4. Materials and Methods

Porcine mucosal heparin, heparan sulphate and chondroitin sulphate were purchased from Celsus (Cincinnati, OH, USA). Heparinases I, II, and III used in the large-scale depolymerization were obtained from Grampian Enzymes (Aberdeen, UK). Chondroitinase ABC was from IBEX (Montreal, QC, Canada). All other reagents used were of analytical grade. The alkaline protease mixture, alcalase, was from Novo (New York, NY, USA). Spectrapore dialysis membranes with a molecular cut-off weight of 1000 Da were from Spectrum Medical (Los Angeles, CA, USA). A small Polyprep column from BioRad (Watford, Hertfordshire, UK) was packed with 500 µL of Diethylaminoethyl DEAE Sephacel (GE-Healthcare, Visalia, CA, USA).

### 4.1. Extraction of Whelk GAGs

Crude GAGs were extracted as a white powder from the shellfish common whelk (*Buccinum undatum*), which was obtained from a local fish market in Manchester (the original collection place was the Irish Sea). The shell was removed and 200 g of the soft body was defatted using 100% acetone for 72 h. The sample was dried at room temperature for 48 h. A blender was used to convert the fat-free dried whelk into in to a powder. Following the extraction method from Kim, et al. [19], 4 g of the powder was suspended in 40 mL of 0.05 M of sodium carbonate buffer at a pH of 9.2, before 2 mL of alcalase enzyme was added to degrade protein and release GAG chain. This step takes approximately 48 h at 60 °C, with constant agitation at 200 rpm. The mixture was cooled at 4 °C before 5% (*w*/*v*) trichloroacetic acid was added to the sample and mixed to precipitate peptides. After 10 min, the mixture was centrifuged at 8000× *g* rpm for 20 min. Three volumes of 5% (*w*/*v*) potassium acetate in ethanol were added to one volume of supernatant, and the solution was stored at 4 °C overnight before being centrifuged for 30 min at 8000× *g* rpm. The precipitate, which was approximately 1 g, was dissolved in 40 mL of 0.2 M of NaCl solution and centrifuged at 8000× *g* rpm for 30 min with the supernatant subsequently discarded. Cetylpyridinium chloride (0.5 mL of 5% (*w*/*v*) solution) was added to the supernatant followed by centrifugation to recover the precipitate, which was subsequently dissolved in 10 mL of 2.5 M of NaCl. Finally, 5 volumes of ethanol were added followed by centrifugation for 30 min at 10,000× *g* rpm. After this, the precipitate was collected and dialyzed against water for 72 h. The water was changed twice daily, before the sample was lyophilized to obtain a white powder containing approximately 2.0 mg of crude GAGs.

### 4.2. Anion-Exchange of Intact GAGs

A small polyprep column from BioRad was packed with 500 µL of DEAE-Sephacel (GE-Healthcare) and the bed was calibrated with 5 mL of diethylaminoethyl DEAE pre-wash buffer (50 mM NaOAc/150 mM NaCl with 0.1% Triton X-100 at a pH of 6.0). A total of 1 mg of crude GAGs was dissolved in 500 µL of deionized water, with 250 µL of this solution was loaded onto the column. After this, the column was washed with 5 mL of DEAE washing buffer (50 mM NaOAc/150 mM NaCl with at a pH of 6.0). The GAGs were eluted by adding 2.5 mL of DEAE elution buffers (1–50 mM NaOAc/1M NaCl pH 6.0, 2–50 mM NaOAc/2M NaCl pH 6.0 and 3–50 mM NaOAc/3M NaCl pH 6.0). Each fraction was collected separately. All samples had salt removed using the PD-10 column.

### 4.3. PD-10 De-Salting Technique

The PD-10 column from GE-Healthcare was used for desalting and buffer exchange. The sample preparation in the range of 1.0–2.5 mL by gravity flow was carried out. The desalting capacity was greater than 90%, and the recovery rate was in the range of 70–95%. The bed dimensions were 14.5 × 50 mm, and the column was prepacked with Sephadex G-25 medium. The column was prepared by washing 6 times with 10% ethanol, before 1 mL of sample was loaded and washed with 2.5 mL of 10% ethanol. The first 2.5 mL of eluted buffer was discarded as it is the void volume of the column. The next 3.5 mL of 10% ethanol were collected and lyophilized.

### 4.4. Enzymatic Degradation of Ion-Exchange Fractions

A total of 100 µL (10%) of each fraction (CK-1, CK-2, and CK-3) was incubated with a mixture of heparinases (30 mU Hep I + 16 mU Hep II+ 16 mU Hep III) and chondroitinase ABC (1.5 µL of 5 IU/µL AC + 1.5 µL of 5 IU/µL B) for 24 h. The same protocol was applied to the standard commercial porcine HS and CS.

All samples were fractionated using a 10,000 MWCO filter (Pall Laborotary/Westborough, MA, USA) (Nanosep centrifugal with an omega membrane). The filter contains the omega membrane that has low protein-binding, modified polyethersulfone on a polyethylene substrate. The filtrate receiver is polypropylene, for rapid purification, separation of mixture and sample fractionation. The membrane pore size is less than 5 nm, and the membrane is able to fractionate biomolecules with a range of 30–90 K molecular weight. To remove the enzyme and undigested GAG chain, the samples were centrifuged at 14,000× *g* for 20 min. The flow-through fragments (the material in the bottom of the Eppendorf tubes) were collected mainly as disaccharides and lyophilized for further analysis. The filter-retained fragments (the material on the top of the filter in the Eppendorf tubes), which was considered to be undigested material, was also dried for further investigation.

### 4.5. Isotopic Aniline Tagging for GAG Disaccharide Analysis by Mass Spectrometry (GRIL-Glycan Reductive Isotope Labeling)

Isotopic aniline tagging for GAG disaccharide analysis by mass spectrometry was used following the protocol described by Lawrence et al. First, 15 µL of ^12^C_6_ isotope aniline and 15 µL of 1 M of 95% sodium cyanoborohydride/sodium (NaCNBH_3_) (Sigma-Aldrich, Poole, UK), was freshly prepared in dimethyl sulfoxide: acetic acid (65:35 *v*/*v*), which was added to each GAG fraction from the ion-exchange chromatography (CK-1, CK-2 and CK-3); after this, 8 pmol of the HS and CS standard disaccharides were used and mixed with the tagging buffer above the tagging buffer. Reactions were carried out at 37 °C for 16 h, before the product was dried in a centrifugal evaporator. After the complete sample was tagged with the ^12^C_6_ aniline isotope label, 17 µL of the LC-MS-grade water (water, 8 mM acetic acid, 5 mM DBA) was added to each fraction (CK-1, CK-2 and CK-3), which was mixed gently to produce a homogenate mixture. The sample was centrifuged at 14,000×*g* for 7 min, before 5 µL was taken and added to LC-MS vials and spiked with 2 µL of 8 pmol internal ^13^C_6_ aniline isotope tag. A total of 1 µL of 10× GRIL buffer and 2 µL of LC-MS water were added to bring the mixture up to the total volume of 10 µL, with only 4 µL injected into the system. A quaternary high-performance liquid chromatography (Thermo-Finnigan, San Jose, CA, USA) was used for disaccharides analyses. Aniline isotopic disaccharides and non-isotopic disaccharides were separated on a C18 reversed-phase column (0.46 cm × 25 cm, Vydac) with the ion-pairing agent dibutylamine (DBA, Sigma-Aldrich). The gradient system used was 100% buffer A (8 mM acetic acid, 5 mM DBA) for 10 min, 17% buffer B (70% methanol, 8 mM acetic acid, 5 mM DBA) for 15 min, 32% buffer B for 15 min, 40% buffer B for 15 min, 60% buffer B for 15 min, 100% buffer B for 10 min, and 100% buffer A for 10 min. Most disaccharides eluted in the 60% buffer B (42% methanol) were further analyzed using the classic quadrupole ion trap mass spectrometer equipped with an electrospray ionization source used for sample ionization and detection. Ions of interest were collected in negative ion mode. The capillary temperature and spray voltage were maintained at 140 °C and 4.75 kV to decrease the in-source fragmentation of sulphated disaccharides.

### 4.6. Quantitative Analysis of Monosaccharides by HPAEC-PAD

The samples containing 1 mg of crude GAGs were dissolved in 1 mL of deionized water, and a 100 µL (10% GAGs) aliquot was treated with 100 µL of 4N trifluoroacetic acid and 4N TFA (prepared by adding 2.40 mL deionized water to 1.00 mL of 13.5 M TFA, providing a stock with 4N TFA) at 100 °C for 4–6 h to cleave all glycosidic linkages, including uronic acids. Following hydrolysis, sample tubes were removed from the heating block and allowed to cool to room temperature. The samples were centrifuged at 27,000×*g* rpm for 3 min to remove any condensation on the side of the tube. Acid was evaporated by dry nitrogen flush for 10 min, and we repeated co-evaporation with 70 µL of 50% isopropanol to remove the acid completely. Finally, the samples were dissolved in 100 µL deionized water and centrifuged, before being analyzed by HPAEC-PAD (Thermo Scientific, San Diego, CA, USA) using a CarboPac PA-1 column with dimensions 4 mm × 250 mm, 4 mm, with a 4 mm × 50 mm Guard. The solvents used were A: Water, and B: 100 mM sodium hydroxide (NaOH) with 5 mM (sodium acetate trihydrate) NaOAc·3H_2_O. The initial condition of 16% B at 1.0 mL/min was applied, a pulsed amperometric detector (PAD) was used in this system. In PAD applications, the electrode is automatically cleaned and prepared for detection by each cycle of the programmed series of potentials (i.e., the waveform), thereby minimizing electrode fouling by oxidation products of the analyte, thus maintaining a consistent response. Monosaccharide standards were treated in parallel and used for calibration of the HPAEC-PAD response.

### 4.7. Routine Cell Culture

Triple negative breast cancer MDA-MB-468 and MDA-MB-231 cell lines were cultured as monolayers. Cells were maintained at 37 °C in a humidified 5% CO_2_ atmosphere. The complete growth medium used to culture the adherent cells were contains Dulbecco’s Modified Eagle’s Medium (DMEM-w/1, g/L glucose without l-glutamine/ BE12-707F) from Lonza, to which 10% FBS (500 mL-FB-1090/500) and 2 mM l-glutamine (100 mL-XC-T1715/100) from Lab Tech (London, UK) were added. Cells were grown in tissue culture flasks T-25 (Vented Filter Cap, TC-Treated-Green-30 × 10-CC7682-4825) from the Star lab to approximately 70–80% confluence, before the media were removed and the cells were washed with warm PBS at a pH of 7.4 (10010023-Thermo fisher scientific, London, UK) prior to trypsinization. The cells were detached by incubation in 1 mL of 1× trypsin-EDTA solution at 37 °C for 5–10 min (Trypsin 0.25%—and EDTA in HBSS without Calcium or Magnesium with phenol red 100 mL of CC-5012) from Lonza (Cambridge, UK). Cells were collected by centrifugation at 1500× *g* rpm for 5 min and resuspended into fresh medium. The medium was renewed 3 times per week from 24 h after each seeding.

### 4.8. MTT Colorimetric Assay

The healthy cells, which had been sub-cultured at least once and subsequently reached 70–80% confluence, were used for the MTT assay. A total of 3000 cells per 100 µL per well were used. After this, the cells were incubated at a temperature of 37 °C with 5% CO_2_. After 24 h of incubation, the cells were examined under the microscope to ensure that there were no signs of contamination and the cells were healthy. Subsequently, 100 µg/100 μL extracts of whelk GAGs, filtered by a 0.2 μm sterile filter, was added into the corresponding wells. The concentration of the extract was prepared in serial dilutions according to the microplate template, which started with a negative control in the first well (row A) until it reached the highest concentration in row H. The assay was performed in triplicate. After this, the plates were incubated to allow the cell proliferation with or without the presence of the whelk GAGs. After 96 h of incubation, the plates were taken out of the incubator and 50 μL of the MTT solution at a concentration of 5 mg/mL was added to each well. The plates were then kept in the incubator for the next 3–4 h until the purple crystals of formazan salt were produced. After this, the medium was removed through gentle aspiration of the liquid, leaving the crystals at the bottom of the wells. Subsequently, 200 μL of DMSO was added to each well to dissolve the formazan crystal, before the absorbance of the formazan solution in the micro plate was measured at 450 nm (OD1), with a reference wavelength at 690 nm (OD2).

## 5. Calculation of EC_50_ Values

The data were analyzed using GraphPad prism 5.0 (GraphPad Software, San Diego, CA, USA). EC_50_ values were determined using a non-linear regression to fit a dose response curve to the data after transforming the drug concentrations to log values on X. The response was normalized and was represented as Y values.

## Figures and Tables

**Table 1:** Disaccharide analysis of crude whelk GAGs. Data are presented as an estimated percentage of each HS disaccharide produced by heparinases I, II, and III digestion.

Disaccharides from Heparinases I, II, and III Digestion of Intact Whelk GAGs	% Mole in CK-1 (1M NaCl) Fraction	% Mole in CK-2 (2M NaCl) Fraction	% Mole in CK-3 (3M NaCl) Fraction	% Mole in Commercial Porcine HS
D0H0	0.00	0.00	0.00	0.00
D0A0	1.70	1.91	10.10	28.35
D0H6	0.12	0.00	0.00	0.13
D2H0	0.23	0.00	0.00	0.03
D0S0	1.85	1.42	7.28	12.39
D0A6	2.19	1.63	1.48	10.55
D2A0	0.79	0.69	0.09	1.33
D2H6	0.36	0.06	0.24	0.41
D0S6	8.83	18.90	16.17	11.21
D0S0	43.15	35.28	28.93	8.81
D2A6	4.18	3.45	0.08	0.98
D2S6	36.60	36.66	35.62	25.81

**Table 2:** Disaccharide analysis of crude whelk GAGs. Data are presented as an estimated percentage of each CS disaccharide produced by chondroitinase ABC.

Disaccharides from Chondroitinase ABC Digestion of Intact Whelk GAGs	% Mole in CK-1 (1M NaCl) Fraction	% Mole in CK-2 (2M NaCl) Fraction	% Mole in CK-3 (3M NaCl) Fraction	% Mole in Commercial Porcine CS
D0a0	4.43	4.02	4.95	3.83
D0a4/D2a0	35.36	20.53	12.21	59.77
D0a6	2.00	2.81	0.67	34.71
D2a4	0.02	0.00	0.00	0.16
D2a0	0.59	1.43	77.74	0.04
D0a10	57.60	71.22	4.42	1.39
D2a10	0.00	0.00	0.00	0.00

**Table 3:** Sulphation types in HS disaccharides in fractions CK-1, CK-2, and CK-3 in whelk GAGs.

Type of Sulphation	HS-CK-1	HS-CK-2	HS-CK-3	Standard Mammalian HS
%Unsulphated	1.70	1.91	10.10	28.35
%*N-*SO_3_	90.44	92.26	88.01	58.21
%*2-O-*SO_3_	85.31	76.15	64.96	37.37
%*6-O-*SO_3_	52.28	60.71	53.59	49.09
Average SO_3_ per disaccharide	2.28	2.29	2.07	1.45

**Table 4:** Sulphation types in CS disaccharides in fractions CK-1, CK-2, and CK-3 in whelk GAGs.

Type of Sulphating	CS-CK-1	CS-CK-2	CS-CK-3	Standard Mammalian CS
%Unsulphated	4.43	4.02	4.95	3.83
%*2-O-*SO_3_	0.61	1.43	77.74	0.30
%*4-O-*SO_3_	92.98	91.74	16.63	61.42
%*6-O-*SO_3_	60.18	75.45	82.84	36.24
Average SO_3_ per disaccharide	1.54	1.69	1.77	0.98

**Table 5:** EC_50_ values of the effect of whelk GAGs fragments produced by heparinases I, II, and III and chondroitinase ABC on TNBC cell lines.

-	Cell Line	EC_50_ µg/mL Crude Whelk GAGs	EC_50_ µg/mL Whelk GAGs + Heparinase I	EC_50_ µg/mL Whelk GAGs + Heparinase II	EC_50_ µg/mL Whelk GAGs + Heparinase III	EC_50_ µg/mL Whelk GAGs + Heparinase I, II, and III	EC_50_ µg/mL Whelk GAGs + Chondroitinase ABC
1	MDA-MB-468	2.80 ± 0.02	2.60 ± 0.01	42.30 ± 0.02	37.90 ± 0.03	33.20 ± 0.01	3.00 ± 0.01
2	MDA-MB-231	3.50 ± 0.01	14.00 ± 0.01	24.90 ± 0.03	26.00 ± 0.03	27.60 ± 0.02	4.60 ± 0.01
